# Job Assessment Through Bioelectrical Measures: A Neuromanagement Perspective

**DOI:** 10.3389/fpsyg.2021.673012

**Published:** 2021-08-12

**Authors:** Margherita Zito, Marco Bilucaglia, Alessandro Fici, Giorgio Gabrielli, Vincenzo Russo

**Affiliations:** ^1^Department of Business, Law, Economics and Consumer Behavior “Carlo A. Ricciardi,” Università IULM, Milan, Italy; ^2^Behavior and Brain Lab IULM, Neuromarketing Research Center, Università IULM, Milan, Italy

**Keywords:** neuromanagement, human resources, EEG, skin conductance, job assessment

## Abstract

During recruitment, human resource departments face two challenges: finding the right people for the job and attracting talent. Therefore, the hiring process requires both the ability to communicate a good company brand image and to understand the characteristics and potential of candidates. In this study, we used a neuroscientific approach to measure the experience of candidates during a job interview. The experiment involved 30 participants that individually took part in a job interview lasting 40 min. During the experiment, their engagement and stress levels were measured in real-time with skin conductance and electroencephalographic (EEG) data. From the results, we identified both the most stressful phases (the second and the fourth parts, relating to the explanation of the job and remuneration) and the most engaging phases (the first and the third phases, relating to the presentation of the company and the explanation of the career process) of the interview, suggesting implications for the assessment process. This study is a contribution to the field of neuromanagement, as a neuroscientific approach was applied to management issues in light of work and organizational psychology.

## Introduction

During the last decades, organizational needs and goals have changed and improved through the Tayloristic methodology, which focuses on new critical elements such as work motivation and the need of an individual to socialize in the workplace (Argentero, 2010). Indeed, the functioning of an organization depends on its working people, and investment in human resources has started to reflect this. As the awareness of what a candidate could contribute to an organization has grown, the assessment process has changed its focus, which has not only become technically oriented but also personality-oriented. These elements require the recruiter to have a new consideration of candidates who are no longer considered “passive” but participatory and able to express ideas, motivations, and goals during the job interview (Schneider et al., 2001), giving more elements to the recruiter who has to evaluate a candidate for a specific role or job, also in light of organizational growth (Abbasi et al., 2020).

Therefore, the selection process is seen as two-way, involving the interviewer and the candidate, with both parties taking an active role in the outcome (Argentero, 2010). If the latter can propose oneself as actively collecting information on the organization and on the job to evaluate the possibility of joining it, the selection process becomes an endeavor that not only seeks to understand the characteristics and potential of the candidates, but also for the interviewer to communicate a good company image. While recruitment specialists have specific strategies for the evaluation of a candidate, the communication of brand image—that is to say, the attempt to “sell” the organization to attract the candidate—actually lacks. This means that the candidate may evaluate their expectations for the new job, also on the basis of previous jobs, by applying a sort of association with what is known that has to be balanced with what is desired. This has important implications for engagement and satisfaction (Davies et al., 2018). From the perspective of the company, this calls into consideration marketing rules and studies suggesting that the familiarity of a brand name has an advantage because of brand awareness, quality, and direct association (Ma et al., 2007). On the basis of these advantages, it is important for an organization that aims to attract talent to show both a good and coherent image of the company and a human resource structure oriented to a practical and positive organization (Schneider and Bowen, 1993; Zito et al., 2019), considering that brand names seem to be saved in the memory of people who are in a structure devoted to associative general knowledge (Keller, 1993). This is in line with the neuroeconomics assumption stating that professional expectations, activities, and relations leave a trace in the functioning of the brain (Cocco, 2016), conditioning subsequent behavior in similar situations.

On the evaluation side, however, emotional activation during a job interview needs to be considered; in fact, public speaking is suggested as a stressor (Sieverind et al., 2005). Moreover, contrary to the traditional assumption that considers job-seekers as rational in the search and choice processes (Kidd, 1998; Emmerling and Cherniss, 2003), other studies highlight the importance of examining the emotional side of these processes (Satpathy, 2012; Bonaccio et al., 2014). This is in line with the assumptions stating that the competitive nature of the selection process can develop negative affective states and stress (McCarthy and Goffin, 2004) and anxiety (Feiler and Powell, 2015; Powell et al., 2018), with the risk of compromising the evaluation of the candidate and his/her performance by the company during the assessment. In this sense, the individual characteristics of the candidate should also be detected, since studies highlight that some personality characteristics, such as conscientiousness, extraversion (Boudreau et al., 2001), and biographical elements, can determine success in a job interview and the ability to manage the evaluation situation (Tay et al., 2006). The issue of personal characteristics in the organizational environment leads also to important considerations on personal resources, positive aspects of the self, links to resilience, and the ability of individuals to control and manage their environment (Hobfoll et al., 2003). These resources can make people able to deal with demanding situations, have a protective role against stress, and allow individuals to perform better (Salanova et al., 2010; Xanthopoulou et al., 2013).

Encouraging the use and expression of such resources could be fundamental for the creation of a friendly and relaxed environment and useful to achieve organizational goals even during the assessment process. From this point of view, one of the resources considered important is self-efficacy. This resource can be useful in the mastery of challenging tasks and in the management of behavior during the job interview (Bandura and Schunk, 1981; Tay et al., 2006), with important consequences on work engagement (Barbier et al., 2012). Therefore, it would be worth considering that a job assessment situation that allows the candidates to experiment with self-efficacy through a relaxing situation—in particular, through a positive and peaceful job interview style—would also allow them to have a better performance. This would make the interview effective because it would allow the organization to detect the potential of the subject.

As for the evaluation of the reaction of the subject during the assessment situation, neurosciences could be a key element, as it provides a different interpretation between real experienced emotions and the rational side and gives the possibility to study the processing of information considering the role played by emotions (Passyn and Sujan, 2006). This approach finds applications among different fields, such as neuromarketing, neuroeconomy, and neuromanagement. The application of neuroscience to these different disciplines, in particular to consumer neuroscience, allows researchers to delve into and apply neuroscientific tools to detect the decision-making process (Plassmann et al., 2012, 2015; Bazzani et al., 2020). This discipline investigates the antecedents and consequences of behavior, looks at the biological side, and even focuses on the information processing and functioning of attention, memory, and emotion (Yarkoni et al., 2011; Plassmann et al., 2015).

Neuromanagement is a key concept by Prof. Qingguo Ma from the Zhejiang University that integrates economics and cognitive neuroscience with management sciences (Ma and Wang, 2006). The concept is based on a neuroscientific approach applied to management issues to explore behavioral and management processes and analyse brain activity. This allows researchers to understand the mental processes of people when facing management situations, human decision-making, and social behaviors and their influence on the management and economic processes (Parincu et al., 2020). Moreover, the neuroscientific approach allows for the reliable detection of emotions and mental processes also linked to self-regulation and social ability in several contexts, such as the professional one (Balconi and Salati, 2020).

According to studies on general consumer behaviors and decision processes, measurements based on the registration of neuro-physiological parameters could give accurate and reliable results because of the fact that they lack the mediation of cognitive processes (Poels and DeWitte, 2006; Missaglia et al., 2017). In fact, neuroscience, when applied to marketing issues, aims to discover what is happening in the brain in response to products or advertising stimuli and, in turn, discover which strategy can lead to the buying process (Ciceri et al., 2019; Russo et al., 2020).

Neuroscience techniques, which are focused on forms of interpretations of reality based on cognitive schemes and experienced emotions and on the detection of decision-making process, can be used and applied in any communication exchange activating a reaction that can be detected (Plassmann et al., 2015; Cocco, 2016; Bazzani et al., 2020). This approach can also help the specific assessment process by measuring reactions useful for the choosing and meeting of organizational goals, detecting those elements related to engagement and stress, and supplying organizations with instruments to evaluate and conduct effective job interviews and, thus, meet their own organizational goals. In fact, being aware of the possibilities offered by recruiting strategies is crucial, since recruiting behavior can have implications in the design of labor market policies (Behrenz, 2001).

Taking together the aim of the research and the used techniques suggest that the meeting between the company needs of brand communication and recruitment evaluations is possible by joining the neurosciences science applied to management issues. In fact, neuromanagement in particular detects how communication can affect people within organizational and work situations (Venturella et al., 2017) through neuroscience instrumentation.

The main aim of this study is to measure the experience of the candidates during a job interview through a neuroscientific approach. The experiment provided a job interview with five different interviewers (A, B, C, D, and E), each belonging to one of the two main interview styles proposed in the literature (Raccanello, 2015; Argentero, 2016). These styles have been further exposed in the hypotheses section: one is characterized by many questions and is potentially stressful, and the other is a quiet style, also characterized by warmness and humor. Interviewers A and E belonged to the first category, while B, C, and D to belonged the second one. The interviews were structured job interviews and included four main phases:

- phase 1, the “ice breaker” and company presentation (namely, P1);
- phase 2, explanation of the work (namely, P2);
- phase 3, explanation of career possibilities (namely, P3);
- phase 4, agency mandate, with the specification of variable remuneration salary (namely, P4).

The four phases are expected to be linked to different engagement and stress levels, as mentioned in the hypotheses section.

To measure engagement and stress levels, we adopted two of the most widespread neuroscientific instruments in marketing research: the electroencephalogram (EEG) and skin conductance (SC) measurements (Alvino et al., 2020). The EEG measures the time-varying electric potential associated with the activity of a large number of neurons within the brain cortex. A differential activity between the left and right frontal cortices (the so-called frontal asymmetry) has been associated with emotional valence: the left frontal cortex is involved in experiencing positive emotions, while the right is involved in processing negative emotions (Davidson, 2004). Frontal asymmetry is defined in terms of frontal alpha asymmetry (FAA) since the electrical power in the alpha band (8–12 Hz) is inversely related to the neuronal activity: a greater left-over-right frontal asymmetry corresponds to a greater right-over-left frontal alpha prefrontal asymmetry and vice versa (Reznik and Allen, 2018). It has to be considered that emotions are defined as an adaptation to problems, and analyses on this topic should consider problematic situations as causes and/or consequences of emotions (Keltner and Gross, 1999). A positive valence underscores an approach toward the stimulus or an interest; on the other hand, a negative valence underscores avoidance toward the stimulus or a detachment (Harmon-Jones et al., 2010). The interest or detachment levels make it possible to understand the level of engagement toward the situations, as engagement is defined as a positive state of mind capturing the experience of a situation (Schaufeli and Bakker, 2010; Bakker and Albrecht, 2018). Moreover, a recent study also underscored the activation of prefrontal areas during the presentation of an advantage for the subject (Balconi and Fronda, 2020), an aspect considered linked to engagement. In this study, we operationalize the FAA as an indicator of engagement.

Skin conductance refers to the electrical conductance of the skin as a consequence of the sympathetic activity of the autonomic nervous system (ANS) on sudomotor nerves. Sudomotor nerves modulate the amount of sweat produced by the sweat glands, decreasing the electrical resistance of the sweat ducts and, in turn, increasing the net conductance of the skin. The skin conductance (SC) signal is modeled as the sum of two distinct components: the SC level (SCL), reflecting slow drifts, and the skin-conductance response (SCR), reflecting fast changes (Posada-Quintero and Chon, 2020). Scientific literature correlates SC and emotions, suggesting SC as a good indicator of arousal (Bolls et al., 2001; Ravaja, 2004; Gakhal and Senior, 2008; Sequeira et al., 2009) and stress or mental workload (Jacobs et al., 1994; Liapis et al., 2015; Greene et al., 2016). In particular, the phasic SCL has been previously adopted for measuring stress levels (Borghini et al., 2020). Accordingly, we operationalize the SCL as an indicator of stress.

## Experimental Hypotheses

According to the main aim of the study, three hypotheses were developed following the neuromanagement perspective from both theoretical and empirical standpoints. Also considering the job perspective, the hypotheses were formulated in light of the literature regarding work and organizational psychology. It has to be noted that the first two hypotheses were particularly related to the phases of the interview, while the third one was related to the style of the interviewer.

This study operationalized the FAA as an indicator of engagement. From a job perspective, engagement is defined as a positive work-related state of mind that can capture how people experience a job situation (Schaufeli and Bakker, 2010; Bakker and Albrecht, 2018). In this view, a job becomes something that stimulates people by creating meaning and positive accomplishments. Linked to engagement assumptions, the assessment framework must also consider expectations (in terms of environment and advancements) of the future in a company, which is also a form of psychological contract (De Vos et al., 2009).

Hypothesis 1: The phases with higher levels of engagement are those related to the ice breaker and company presentation (P1) and career (P3).

If, on one hand, this study considered the most engaging phases, the research also assessed the most stressful parts of the interview with the operationalized SCL. Stress occurs in work situations and the main antecedents of job stress are related to the role in the organization, the career possibilities, the organizational and the distribution of the tasks structure (Cooper and Marshall, 1978), but also the job demands of an organization (Bakker and Demerouti, 2014), with consequences linked to exhaustion (Setti et al., 2018) and to the turnover intentions (Hallin and Danielson, 2008). This perspective has to be particularly considered in the variable remuneration rules explained in the fourth phase of this study since it could be linked to the perception of job insecurity, which can then make a job less appealing and become a source of perceived stress (De Witte, 1999; Witte et al., 2015). In order to avoid this scenario and in light of the anticipatory psychological contract, detecting this variable is important in the assessment step.

Hypothesis 2: The most stressful phases are those related to the explanation of the job (P2) and remuneration (P4).

Beyond observations on the engagement and stress indices, this study also explored the possible effect of the type of interview on the candidates. According to previous studies, the way of interaction between the interviewers and the candidates can be influenced by certain individual variables, in particular, quiet and positive moods that can strongly influence motivation and social behavior (Raccanello, 2015). A quiet style with transmitting positive moods can enhance flexibility, creativity, motivation, and cooperation (Isen and Reeve, 2005; Raccanello, 2015). This point is particularly crucial, considering that the performance of the candidates can be influenced by his/her perceptions of the situation during the job interview (Melchers et al., 2012). In particular, the affective approach during interviews, characterized by warmness, agreement, and humor, can contribute to the reduction of the anxiety of the candidates (Carless and Imber, 2007), whereas a competitive and pressing situation can lead the interviewed subject to anxiety and stress (McCarthy and Goffin, 2004). Furthermore, an effective approach can be characterized by the personalization level of communication style (Scheuer, 2001); that is, the inclusion of elements linked to the self or expressing personal feelings or experiences, thus establishing a relationship between the interviewer and the candidate. Accordingly, this would put the candidates who feel warmness, more commitment, and encouragement in the personal interaction at ease (Scheuer, 2001; Raccanello, 2015).

Among other theoretical standpoints, the way of conducting an interview can be based on different styles (Argentero, 2016). The first can be defined as “friendly,” in which the power of the exchange is equally distributed. This style is not always the best choice in job assessment, since the interviewer needs to conduct the interview. Another style is based on a professional exchange based on assertiveness. In this case, questions and answers alternate, and both interlocutors are involved in a positive atmosphere of mutual openness, with the aim of creating a relaxed situation based on respect and trust. Finally, another style can be based on the so-called “stress interview.” In this case, the interview involves a series of questions (a sort of “interrogation”) posed in a direct and even intrusive way, for which precise answers are expected, in a strongly structured situation where interaction is exclusively guided by the interviewer. This results in a tense and uncomfortable atmosphere, which was created to understand how the candidate can react in stressful situations but not allowing the candidate to express his/her potential and, moreover, not allowing the organization to understand the real competencies and skills of the candidate.

On the basis of the above-mentioned theoretical standpoints, we assume that a cooperative and positive atmosphere would be more productive and favorable for both the organization and the candidate. On one hand, studies highlight the need for environments that can put their candidates at ease (Argentero, 2010), also in light of the possibility for these candidates to feel positive emotions and perform well (Demerouti, 2006; Zito et al., 2019). On the other hand, studies also focus on the crucial role of the support received by the candidate during the job interview as a resource that can decrease the level of stress (Frisch et al., 2014, 2015). Assuming these theoretical points of view, we operationalized the conduction styles as shown in the following method section. It is, therefore, hypothesized that:

Hypothesis 3: A calm and peaceful style of interview (B, C, and D) will produce less stress in the candidate.

## Materials and Methods

### Sample and Procedures

This study has been conducted in the specific framework of assessments: a large Swiss company involved in the energy market made available its standardized interview, which is used to assess candidates looking for a job in the company. This company is very solid in the European labor market; therefore, this study considered the assessment interview as a consistent basis to test a novel neuroscientific approach for human resources.

The participants were real candidates who applied for a real job interview. They were informed about the experiment, and they voluntarily accepted to perform their assessment within the laboratory conditions. There were 30 participants (13 males; 17 females) with an age range of between 25 and 45 years (M = 35.23, SD = 8.05) and were both high school and university graduates looking for a job. All the participants signed an informed consent before the experimental procedure began. None of the participants reported any history of brain disorders or neurological surgery at the date of the enrolment.

In order to avoid a possible carry-over effect, the participants were randomly assigned to the different interviewers that the company assigned to the assessment. Six candidates were assigned to each interviewer. The interviewers were chosen by the energy company, and they belonged to that organization. The researchers could not intervene in the choice of interviewers.

After entering the laboratory, the researchers explained to the participants all the procedures and the phases they were going to experience. In order to track real-time engagement and stress levels, the participants wore two lightweight and wearable devices for EEG and SC measurement. The setting reflected an assessment situation with the interviewer and candidate sitting and facing each other.

A 60-s long baseline phase (P0) was recorded before the beginning of the interview. The subjects were not given any particular instructions, except for trying to relax as much as possible while both the EEG and SC basal activities were recorded according to previous studies (Bilucaglia et al., 2019; Gabrielli et al., 2020; Laureanti et al., 2020; Russo et al., 2021).

As for the style of the interviewers, according to the above-mentioned theoretical standpoints on the type of interviews and on the basis of conducting style of the interviewers participating in the research, we operationalized two main categories of styles. We provided an observation that considered the method of interviewing and then a classification of the style on the basis of the observed characteristics (Mason, 1994; Jamshed, 2014). This classification followed the characteristics shown in the literature in terms of styles and consequences on the candidates. Going more in-depth, we identified two main styles: the first is characterized by a positive atmosphere with a quiet and peaceful style aimed at putting the candidate at ease through warmness, humor, and openness (Isen and Reeve, 2005; Raccanello, 2015). This type of style was associated with interviewers B, C, and D. The second style is characterized by a lot of questions in a short time and in a competitive and stressful atmosphere (McCarthy and Goffin, 2004; Argentero, 2016). This type of style was associated with interviewers A and E.

### Instrumentation

The electroencephalogram data were recorded using B-Alert X10 (ABM Inc. New York, NY, United States), which is a wearable and wireless headset. It has nine Ag/AgCl wet electrodes (arranged in a monopolar montage) embedded in a flexible plastic strip, located at the F3, Fz, F4, C3, Cz, C4, P3, POz, and P4 sites of the 10/20 system (Jasper, 1958). The device allows the linked mastoid reference based on two Ag/AgCl adhesive patches placed on M1 and M2. The sample frequency is 256 Hz, and the resolution is 12 bits (Hairston et al., 2014). Prior to the application of the Synapse^©^ conductive cream (Kustomer Kinetics Inc., Arcadia, CA, United States), the skin was properly scrubbed with 70% isopropyl alcohol in order to reduce electrode impedance. The SC signal was recorded using the Shimmer 3 GSR+ (Shimmer Sensing Ltd., Boston, MA, United States), which is a wearable and wireless bracket-like device. According to the recommendations in the literature (Boucsein et al., 2012), SC was recorded using a constant-voltage mode (0.5 V) by means of two Ag/AgCl electrodes placed on the index and ring fingers of the non-dominant hand.

Both the electroencephalogram and SC wireless data streams were collected with a personal computer (PC) and synchronized using iMotions v.6.1 (iMotions, A/S, Copenhagen, Denmark), an integrated software research platform. The interviews were recorded using a LifeCam Studio webcam (Microsoft Corporation, Redmond, WA, United States), and the video was real-time synchronized to both the electroencephalogram (EEG) and SC data by iMotions. The video recordings served to segment each interview into the above-mentioned phases (P1, P2, P3, and P4).

### Data Processing

The EEG data were processed using iMotions and B-Alert software development kits (SDKs). First, the decontamination procedure illustrated by Berka et al. (2007) was applied. Noise (e.g., spikes, high voltage excursions) and artifact (e.g., eye blinks, EMG noise) data points were identified by means of either a wavelet transform or thresholds on both the amplitude and the time, as well as using a linear discriminant analysis classifier. Depending on their type (i.e., noise or artifacts), the identified points were either filtered by means of the wavelet transform or simply set to zero.

Then, the decontaminated EEG signal was segmented using a 1-s long Keiser window with 50% overlapping. By means of the B-Alert SDKs, for each frontal channel k, the power spectral density (PSD) was computed within each window and averaged every three windows, obtaining a time signal of concatenated PSDs and PSD_k_(t) with a temporal resolution of 1 s. The alpha powers of F3 and F4 channels, p_F3_(t) and p_F4_(t), were automatically computed by integrating the corresponding PSD_F3_(t) and PSD_F4_(t) in the alpha band (8–12 Hz). A logarithmic transformation was performed in order to mitigate the skewness of the power values. The frontal alpha asymmetry FAA(t) (Reznik and Allen, 2018) was computed as:

$$\begin{array}{l} \text{FAA}\left(\text{t}\right)=\text{log}\left\{{\text{p}}_{\text{F}3}\left(\text{t}\right)\right\}-\text{log}\left\{{\text{p}}_{\text{F}4}\left(\text{t}\right)\right\} \end{array}$$

The obtained FAA(t) was then exported to Matlab R2016 (Mathworks Inc., Natick, MA, United States) and segmented according to the phases of the interview identified by means of video recordings. Within each segment, the outliers were detected using the inter-quantile range (IQR) criteria, as those data points outside the interval [Q1–1.5 × IQR; Q3 + 1.5 × IQR], where Q1 and Q3 are, respectively, the first and third quartiles (Russo et al., 2021). Outlier points, set as “Not-A-Number,” were excluded from subsequent analyses. In order to remove subjective variability (Bilucaglia et al., 2019; Gabrielli et al., 2020), FAA(t) was z-score transformed as:

$$\begin{array}{l} \text{FAA}\left(\text{t}\right)=\;\left(\text{FA}{\text{A}}_{0}\left(\text{t}\right)-\text{m}\right)/\text{s} \end{array}$$

where FAA_0_(t) is the untransformed FAA signal, and m and s are the temporal mean and the temporal standard deviation of FAA_0_(t), respectively, calculated in the baseline phase (P0). Because of the standard deviation in the denominator, the z-score transformation makes any dimensional signals (e.g., the power values expressed in μV^2^ or conductance value expressed as μS) unitless. Finally, z-scored segments were temporally averaged in order to get a condensed phase-related indicator FAA_i_. This procedure was applied on each subject i, giving a set of averaged indicators indexed as FAA_i,j_.

The SC data were exported to Matlab and filtered using a second order low-pass FIR filter (f_c_ = 1 Hz), as a simple procedure to attenuate external noise and large artifacts (Posada-Quintero and Chon, 2020). Then, a low-pass FIR filter (fc = 1 × 10^−12^ Hz, 8,192 samples long) was applied in order to extract the tonic SCL component (Subramanian et al., 2019). Similar to those of the EEG, the SCL data were finally segmented into the interview phases i, cleaned from the outliers using the IQR criterion, z-score transformed, and temporally averaged to obtain a condensed phase-related indicator SCL_i_. The procedure was applied on each subject j, giving a set of averaged indicators, indexed as SCL_i,j_.

### Statistical Analyses

Statistical analyses were performed using JASP v.0.14 (Love et al., 2019). FAA and SCL indicators were analyzed by two-way mixed ANOVA, considering the interviewer as a between-subject factor (five levels: A, B, C, D, and E) and a within-subject factor the interview phase (four levels: P1, P2, P3, and P4). Prior to the analyses, the sphericity of the phase factor and the equality of variances for the interviewer factor were assessed by the Levene's and Mauchly's tests, respectively (Verma, 2015).

## Results

The results allowed us to identify the most stressful and most engaging phases of the interview, as well as understand which interview strategies can reduce the stress level and improve the engagement of candidates. Table 1 shows the mean scores (M) and the standard deviations (SD) of the indicators (z-score transformed FAA and SCL), split for the four phases of the interview.

**Table 1:** Mean (M) and standard deviation (SD) for the FAA and SCL z-scores, split into the four phases of the interview.

		**P1**	**P2**	**P3**	**P4**
FAA	M	0.502	0.448	0.504	0.363
	SD	0.053	0.044	0.033	0.051
SCL	M	19.999	35.012	25.940	42.838
	SD	8.925	8.261	13.098	8.613

The results regarding the different phases are shown within the following hypothesis sub-paragraphs. Among the results, it is interesting to monitor data along the different interview styles through the general trend. For this reason, many results in the Figures are split within the interviewers.

### Hypothesis 1

For the FAA, both the interviewer main effect and the interviewer × phase interaction were not significant [*F*_(4,25)_ = 1.892, *p* = 0.143, η^2^ = 0.018 and *F*_(12,75)_ = 0.0394, *p* = 0.962, η^2^ = 0.617, respectively], while the phase main effect was significant [*F*_(3,75)_ = 55.254; *p* < 0.001]. *Post-hoc t*-tests with Bonferroni's correction confirmed a significant difference between all the phases (all *p* < 0.001), except between P1 and P3 (*p* > 0.05). The highest FAA values were found in the phases P3 (M = 0.504, SD = 0.033) and P1 (M = 0.502, SD = 0.053) and associated with career possibilities and ice-break company presentation, respectively. The lowest FAA values were found in P4 (M = 0.363, SD = 0.051) and associated with agency mandate, followed by P2 (M = 0.484, SD = 0.044), which was associated with work explanation.

**Figure 1:**
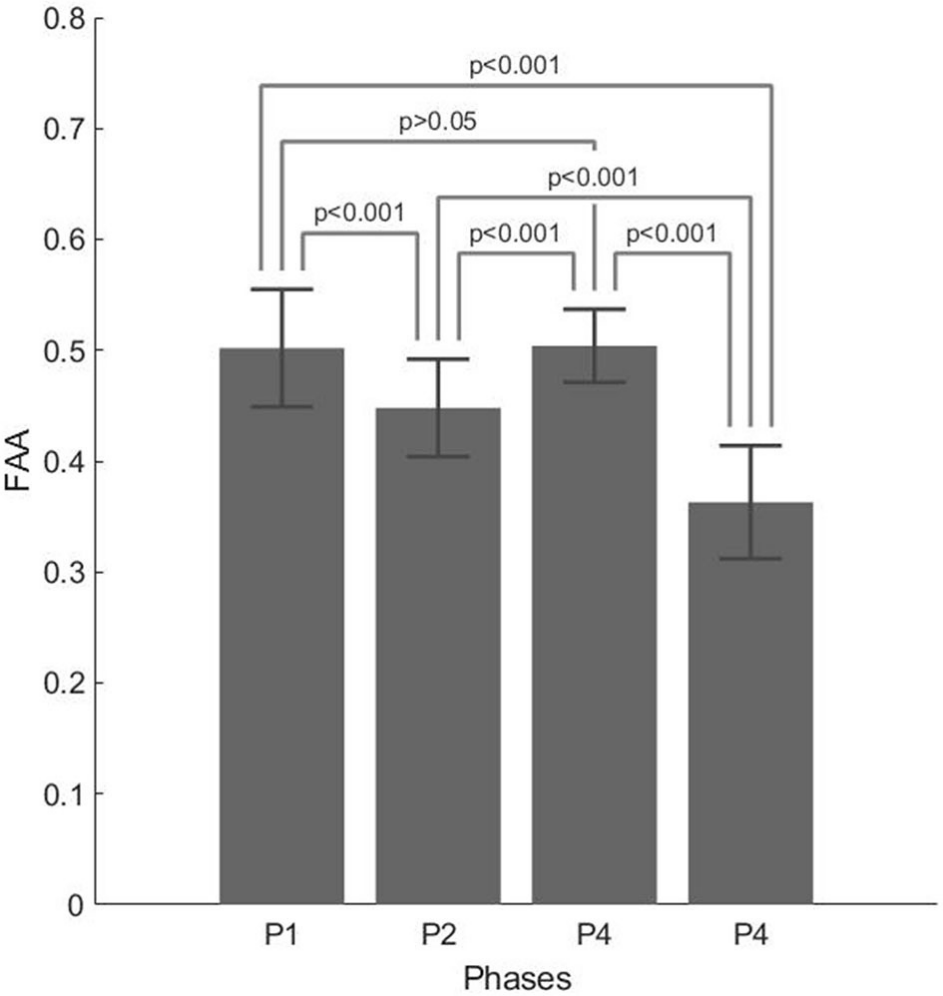
Descriptive plot with error bars for frontal alpha asymmetry (FAA) z-scores split for the four phases of the interview.

Figure 1 and Table 2 report the following, respectively: descriptive plot (with error bars) and descriptive statistics of the EEG data split in the four phases of the interview.

**Table 2:** Mean (M) and standard deviation (SD) of the FAA z-scores, split into the four phases of the interview.

	**P1**	**P2**	**P3**	**P4**
M	0.502	0.484	0.504	0.363
SD	0.053	0.044	0.033	0.051

### Hypothesis 2

For skin conductance level, the interview × phase interaction effect was not significant [*F*_(12,75)_ = 0.778, *p* = 0.671, η^2^ = 0.034], while the phase showed a significant main effect [*F*_(3,75)_ = 40.685; *p* < 0.001, η^2^ = 0.443]. *Post-hoc t*-tests with Bonferroni's correction confirmed a significant difference between all the phases (all *p* < 0.001, except for P1–P2 and P2–P4 where *p* < 0.01). The highest skin conductance level (SCL) values were found in P4 (M = 42.838, SD = 8.613) and P2 (M = 35.012, SD = 8.261), which were associated with agency mandate and work explanation, respectively. The lowest SCL values were found in P1 (M = 19.999, SD = 8.925), the phase associated with ice-break company presentation, followed by P3 (M = 25.941, SD = 13.098), the phase associated with career possibilities.

**Figure 2:**
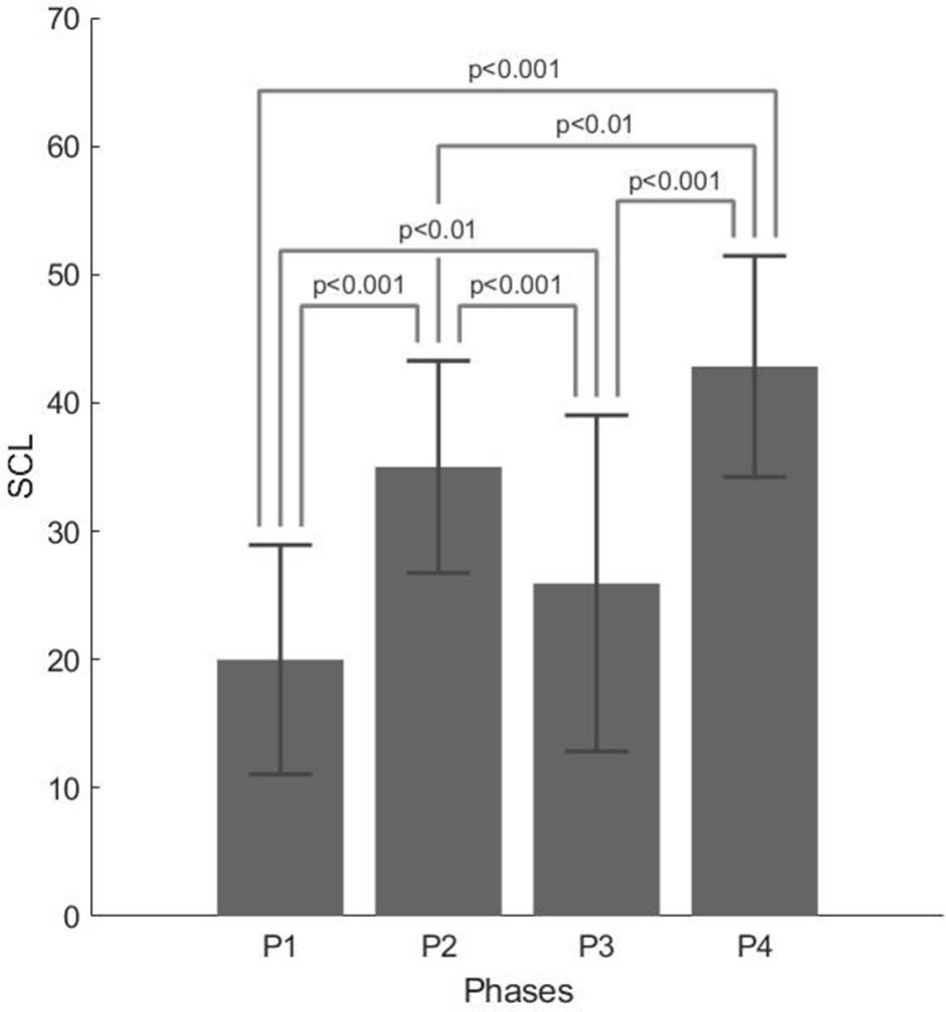
Descriptive plot with error bars for the skin conductance level (SCL) z-scores split for the four phases of the interview.

Figure 2 and Table 3 report the following, respectively: descriptive plot (with error bars) and descriptive statistics of the SC data, split in the four phases of the interview.

**Table 3:** Mean (M) and standard deviation (SD) of the SCL z-scores, split into the four phases of the interview.

	**P1**	**P2**	**P3**	**P4**
M	19.999	35.012	25.941	42.838
SD	8.925	8.261	13.098	8.613

### Hypothesis 3

For the skin conductance level (SCL), the main effect of the interviewer was significant [*F*_(4,25)_ = 11.462; *p* < 0.001, η^2^ = 0.162]. *Post-hoc t*-tests with Bonferroni's correction confirmed a significant difference between A–B (*p* < 0.001), B–E (*p* < 0.001), C–E (*p* < 0.001), and D–E (*p* < 0.01). The highest SCL values were found for A (M = 34.727, SD = 11.155) and E (M = 38.59, SD = 12.729), where the interviewers characterized by a warm and humoristic style. The lowest SCL values were found for B (M = 23.345, SD = 14.195), C (M = 29.652, SD = 11.673), and D (M = 28.422, SD = 11.071), the interviewers characterized by many questions and potentially stressful.

**Figure 3:**
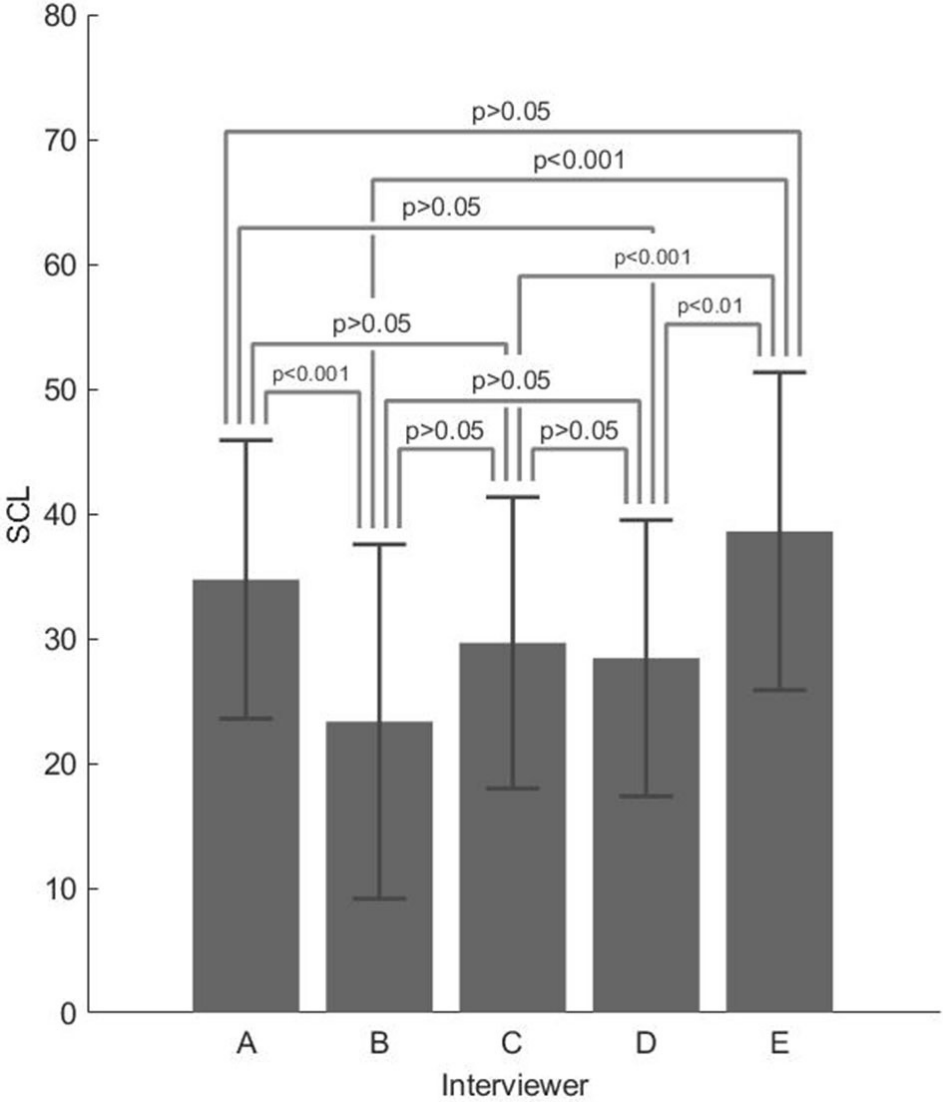
Descriptive plot with error bars of the skin conductance level (SCL) z-scores split for the five interviewers.

Figure 3 and Table 4 report the following, respectively: descriptive plot (with error bars) and descriptive statistics of the SCL z-scores, split among the five interviewers.

**Table 4:** Mean (M) and standard deviation (SD) of the SCL z-scores, split into the five interviewers.

	**A**	**B**	**C**	**D**	**E**
M	34.727	23.345	29.652	28.422	38.590
SD	11.155	14.195	11.673	11.071	12.729

Despite both the interviewer main effect and the interviewer × phase interaction being not significant, the highest frontal alpha asymmetry (FAA) value was found for A (M = 0.469, SD = 0.072), while the lowest one was found for E (M = 0.422, SD = 0.087) where both the interviewers were characterized by a warm and humoristic style.

**Figure 4:**
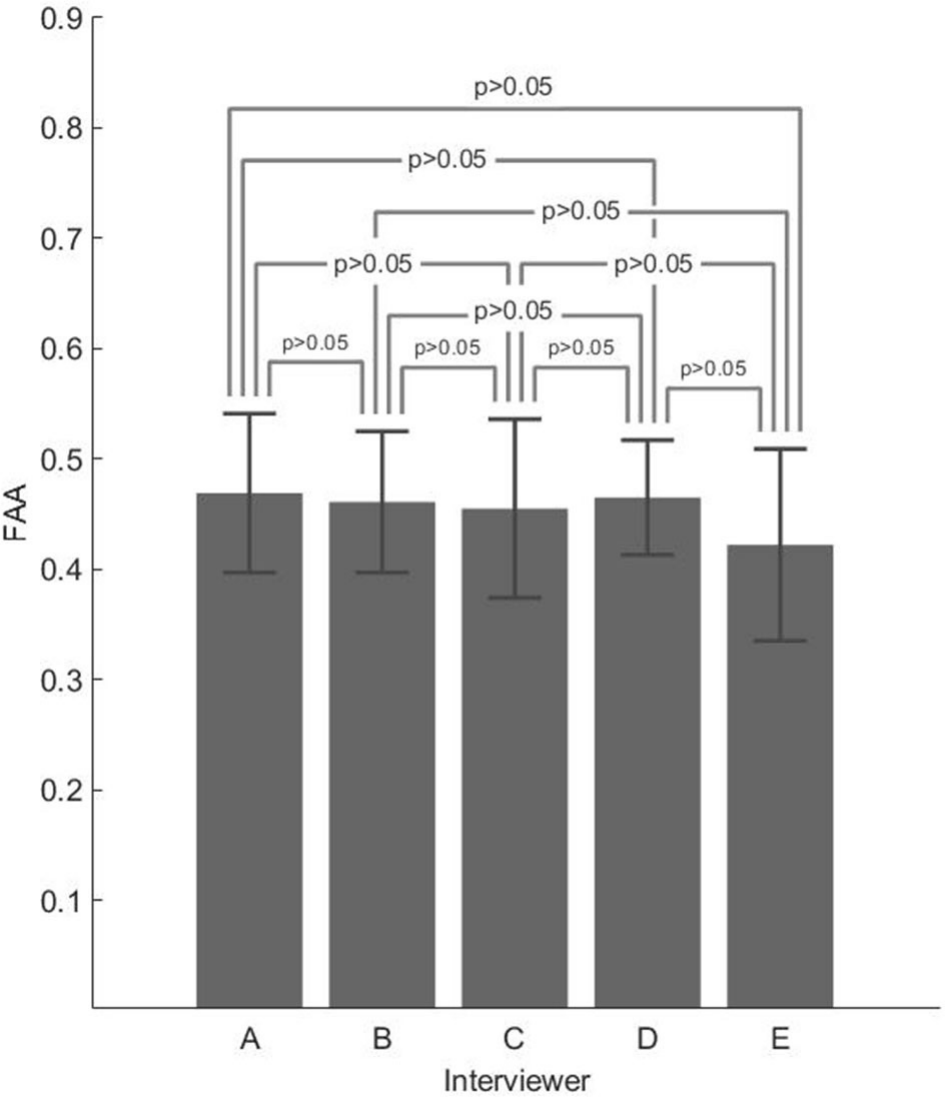
Descriptive plot with error bars of the frontal alpha asymmetry (FAA) z-scores split for the five interviewers.

Figure 4 and Table 5 report the following, respectively: descriptive plot (with error bars) and descriptive statistics of the FAA z-scores, split among the five interviewers.

**Table 5:** Mean (M) and SD of the FAA z-scores, split into the five interviewers.

	**A**	**B**	**C**	**D**	**E**
M	0.469	0.461	0.455	0.465	0.422
SD	0.072	0.064	0.081	0.052	0.087

## Discussion

In this study, we recorded the EEG and SC data of 30 candidates during a 40-min long structured job interview in order to monitor in real-time their engagement and stress levels. The engagement was operationalized through the FAA, and the stress through tonic SCL. The subjects were randomly assigned to five interviewers who differed in interview style: two interviewers were characterized by a style with many stressful questions (A, E) and three interviewers by a more focused, warm, and humorous approach (B, C, and D). The interviews were divided into four phases, characterized by different engagement and stress levels: the “ice breaker” (P1), explanation of the work (P2), career possibilities (P3), and agency mandate (P4). We hypothesized that the different phases would produce different engagement and stress levels (Hypotheses 1 and 2), and that the different interviewers would produce, on average, different stress levels (Hypothesis 3).

Among the results, the FAA showed that the most engaging phases were P1, referred to as the ice-breaking activity and presentation of the company, and P3, related to the explanation of career possibility, confirming hypothesis 1. The FAA was operationalized as an indicator of engagement that the literature suggests to be linked to positive work-related states of mind that capture how people can experience a job (Schaufeli and Bakker, 2010; Bakker and Albrecht, 2018). According to studies, job engagement reflects the creation of meaning associated with the job and, from the perspective of the candidates, these aspects, referred to as the expectations on the environment and on what a future in the company could be, appear relevant from a development standpoint. This point, indeed, is defined as crucial in assessment dynamics, since expectations are seen as an anticipatory psychological contract; that is, the belief of individuals in the employment also concerns guarantees to the future employer and expected inducements (De Vos et al., 2009), and is functional to psychological empowerment and employee engagement in future organizational actions (Sandhya and Sulphey, 2019).

On the side of skin conductance level (SCL), data showed that P1, the phase related to the ice-breaking activity and company presentation, is characterized by less stress. Read together with the higher level of FAA measurement, it could lie on the possibility for building a new perspective, collecting information about the company, and evaluating what the guarantee of the candidates could be. These results are also linked to the possibility of experimenting with the so-called “good stress” (Karasek et al., 1998), which implicates the development of active behaviors when demanding situations that are in line with the possibility to decide, therefore leading to motivation or new learning behaviors. In contrast to this first more relaxed phase, SCL underscores that P2, related to the explanation of the job, and P4, referred to as agency mandate with the specific characteristics of the VAT number and explanation of work with variable remuneration, are the most stressful for the candidates. These results, confirming hypothesis 2, could be related, on one hand, to the effort to understand the organization of the job and distributions of the role applied for (Cooper and Marshall, 1978; Setti et al., 2018). On the other hand, the results on P4 could be related to the fact that variable remuneration provides a high effort or unknown reward outcomes. Indeed, remuneration, beyond career satisfaction, is considered a crucial work value in determining career success, which often makes candidates expect high financial rewards (Dries et al., 2008). Variable remuneration is also linked to the issue of duration of the work contract and, therefore, job insecurity, which is a crucial and discussed topic in the field of work and organizational psychology. The psychological concept of job insecurity refers to concerns about the fear of losing a job and becoming unemployed (Rothschild and Hyun, 1990; Sverke and Hellgren, 2002). Job insecurity, indeed, is conceptualized as a source of stress (De Witte, 1999; Witte et al., 2015), with detrimental effects on the well-being of employees and their psychological and physical health (Sverke and Hellgren, 2002; Emberland and Rundmo, 2010; Laszlo et al., 2010; Kerse et al., 2018). Therefore, when evaluating a job situation, this point has to be considered in the enhancement (even reduction) of stress levels.

As for the styles of the interviewers, even if this is the first step of the research, the style based on many questions to the candidates (A and E) produced more stress; on the other hand, the quiet and peaceful style (B, C, and D) seemed to produce less stress in the candidates, confirming hypothesis 3. The non-significant results for the FAA suggest that the style of interview does not affect the engagement of the candidate. The results are in line with the two main styles that we operationalized. At this point in the research, this could be linked to the influence of effect on job interviews (Raccanello, 2015). In fact, a competitive selection can foster the rising of negative affective states, such as stress, frustration, and anxiety (McCarthy and Goffin, 2004), even compromising the performance of the candidates but also reducing their ability to adopt successful strategies (Bonaccio et al., 2014). On the other hand, the affective approach of the interviewers, characterized by warmness, agreement, and humor, can have a role in reducing the anxiety of candidates (Carless and Imber, 2007). Moreover, as already highlighted, it is important to consider the role of support (also in terms of a supporting atmosphere), even coming from the interviewers, since it could be functional in decreasing the levels of stress among candidates (Frisch et al., 2014, 2015). On the basis of the conduction style, it is suggested to deepen expectations and the aspirations from the beginning, referring to the experiences, passions, and hobbies of the candidates. Moreover, it is suggested to encourage the candidates to share these aspirations, leaving space for their stories, to understand the attitudes and qualities of the person, and to put the candidate at ease in order to enhance their self-efficacy (Consiglio et al., 2016). Starting from the result of this study, it is important to present company qualities, especially from an economic-financial point of view with a long history behind it. Underlining its uniqueness and linking it to a complete offer of products and services could be strategic to sell the company image and communicate a good company brand image (Ma et al., 2007); it may also be useful for positive evaluation from the candidates. Moreover, focusing both on the expectations of the candidates and on the presentation of the company is useful to prevent the presence of people looking for a job without any awareness or with haphazard job search strategies (Bonaccio et al., 2014).

As for stress produced by those several questions that are necessary to know the characteristics, attitudes, and expectations of the candidates, it could be useful to offer resources, such as support from supervisors or the possibility to have job autonomy, in line with the Job Demands-Resources Model (Bakker and Demerouti, 2014). Considering not only the demands but also the resources that an organization could make available for employees could reduce distress outcomes and foster well-being. This can also be done through positive experience at work, which is functional to protect from emotional or cognitive and physical exhaustion (Zito et al., 2016). In fact, a situation of strain can also occur when the psychological demands are higher than the possibility to use a very important resource, such as autonomy (Zito et al., 2019) or decision latitude (Karasek and Theorell, 1990).

As the psychological contract plays a relevant role (De Vos et al., 2009), it could also be useful to create an adequate interview on the expectations to meet the needs of the company and the candidate, in order to better reach organizational goals.

Neuromanagement is quite a young discipline, and this study would contribute to deepening this issue in light of organizational psychology. In line with recent guidelines and studies on neuromanagement, it has to be underscored that this discipline can design new intervention strategies that can develop a direct evaluation of both individual potential and performance (Parincu, 2019; Balconi and Salati, 2020) with a positive outcome on employee growth and organizational efficacy. As neuromanagement deepens the research on this subject, it is important to consider that this is the first discipline to look at an organizational issue considering the brain processes deriving from the dynamics in an organization and allowing the detection of human brain actions and interactions in the business context from the neuroscience perspective (Ghadiri et al., 2012). In this sense, neuromanagement can be useful for the emotional brain and building of social connections, helping organizations in managing emotions in the workplace (Parincu, 2019), and preventing negative emotional dynamics and designing positive emotional and collaborative workplaces. This would be particularly useful in the perspective of assessment, in which it is important to detect the real aspect and potential of candidates. In light of the potential of the individual, the possibility to use neuroscience tools during the assessment session could be functional in better capturing the abilities and qualities of an individual. This would match important organizational aims such as informing employees of their future perspectives and possibilities or advising employees on the job to improve career opportunities (Balconi and Salati, 2020). This is an interesting point to deepen and to take into account that this study has also confirmed with the high engagement of candidates found in P3, which is related to the explanation of career possibilities. In this sense, even if this is a first pilot study, it uses a neuroscientific approach that can be a first contribution to understanding their abilities and potential. Understanding the engaging and stressful elements in this crucial phase is very important to select the right person and to understand the phases to be activated for their growth, even in terms of organizational performance. Moreover, having participatory and engaged candidates is functional for organizations to communicate the characteristics and all the crucial information needed to “sell” the job to the ideal candidate.

Even if this is a first exploratory study, as listed, it calls into consideration different implications and practical suggestions for human resources departments engaged in the assessment process. Knowing the psychological and physiological implications of the different phases of the hiring dynamics not only allows companies to attract and identify the right employees but also to plan future organizational implications. In fact, involved and motivated individuals aware of the challenges and possible available resources can contribute to the creation of positive organizational environments (Zito et al., 2016). This is functional to enhance motivation and give meaning to the job with positive consequences on performance and on individual and collective well-being (Demerouti, 2006; Cantele, 2018), also in light of the importance of investing in human resources management policies related to human capital and the evaluation and enhancement of the resources of the individuals (Manuti et al., 2020) that are identified as competitive advantages for an organization.

The limitation of this study is related to the detection of a unique work context. This did not permit the generalization of results, but it gives suggestions for the management of job interviews in pursuit of reaching organizational goals. Even with this limitation, however, this study shows an important strength, since it uses neuroscience techniques that allowing the capture of real reactions in real-time, which is useful in declining new assessment and evaluation directions. In fact, at the implication level, the ability of the interviewers to adapt to the candidate and to create empathy was crucial, though an in-depth analysis of personal aspects and life experiences was not strictly linked to a mere work aspect. Therefore, applying the neuroscience approach to strategic company planning that considers both the internal organization and the external communication appeared to be a key factor, and it allowed it to flow into the issue of neuromanagement. This approach can also be useful to explore organizational processes and the way to react and cope with daily job circumstances (Venturella et al., 2017). Moreover, the fact of collecting data in real-time allows us to have data on real reactions, not mediated by cognitive elaborations typical of the retrospective approach. Another limitation of the study is the classification of the characteristics of the interviews only on the basis of observations. Future studies should classify these characteristics (such as humorous or stressful cadencies) also considering the characteristics, traits, and expertise of the interviewers. A limitation of this study is also linked to large standard deviations, particularly for the SCL data. This could be linked to the different characteristics of the candidates composing the sample (Van Voorhis and Morgan, 2007), and future studies should also consider and detect these aspects.

For the future, this study should collect more data among different working categories in order to try to generalize data, and to extend and detect critical phases among other working contexts and cultures. Moreover, age should be useful in the investigation of the stress reactions that could be particularly highlighted within young job-seekers, since they would be less able to manage emotions because of their inexperience in job assessment (Bonaccio et al., 2014).

Furthermore, interesting points for the future could be the detection of possible differences between women and men and the analysis if the level of salivary cortisol before, during, and after job interviews, which would help researchers understand and correlate hormone fluctuations among different steps and interview phases with stress and activation reactions. This could be in line with studies indicating different gender approaches to risky decision-making, which seem to be influenced by different levels of stress hormones (van de Bos et al., 2014). Finally, future studies should replicate this study by also taking into account the reactions of the interviewers, investigating, e.g., the bioelectrical signal similarities with the candidate (Gabrielli et al., 2020). According to the emotional contagion assumptions (Hatfield et al., 1993), people tend to mimic facial or vocal expression, postures, and behaviors of people around them; thus, also acquiring their emotions. This could be functional in detecting how the interviewer can influence the candidate, and suggests the need for specific training to conduct assessments that are functional to put the subject at ease. This is also in line with theory about the “chameleon effect” (Tanner et al., 2008), which suggests that people tend to unconsciously imitate the body movements and facial expressions of others, also in order to facilitate the emotional connection. This effect could also be used to condition the behavior of others: it could be interesting that the awareness of a trained interviewer can be used to positively affect the mood of a candidate in order to reduce stress levels and increase his/her performance, allowing the company to conduct a more precise selection process.

## Data Availability Statement

The raw data supporting the conclusions of this article will be made available by the authors, without undue reservation.

## Ethics Statement

The studies involving human participants were reviewed and approved by Ethics committee of Università IULM. Participants provided their written informed consent to participate in this study.

## Author Contributions

VR and GG designed the research. MB and AF collected the data and carried out data analysis and interpretation. MZ and MB wrote the manuscript. MZ, AF, and VR edited the final version. VR and GG supervised the project and the writing of the study. All the authors contributed to this study, final version of the manuscript has been approved for submission, and accountable for the whole study.

## Conflict of Interest

The authors declare that the research was conducted in the absence of any commercial or financial relationships that could be construed as a potential conflict of interest.

## Publisher's Note

All claims expressed in this article are solely those of the authors and do not necessarily represent those of their affiliated organizations, or those of the publisher, the editors and the reviewers. Any product that may be evaluated in this article, or claim that may be made by its manufacturer, is not guaranteed or endorsed by the publisher.

## References

[B1] Abbasi S. G. Tahir M. S. Abbas M. Shabbir M. S. (2020). Examining the relationship between recruitment & selection practices and business growth: An exploratory study. J. Public Affairs 21:e2438. 10.1002/pa.2438

[B2] Alvino L. Pavone L. Abhishta A. Robben H. (2020). Picking your brains: Where and how neuroscience tools can enhance marketing research. Front. Neurosci. 14:577666. 10.3389/fnins.2020.577666 33343279 PMC7744482

[B3] Argentero P. (2010). La selezione del personale, in Psicologia delle risorse umane, eds Argentero P. Cortese C. G. Piccardo C. (Milano: Raffaello Cortina Editore). 17–48

[B4] Argentero P. (2016). L'intervista di selezione. Teoria, ricerca, Pratica. Milano: Franco Angeli.

[B5] Bakker A. B. Albrecht S. (2018). Work engagement: current trends. Career Dev. Int. 23, 4–11. 10.1108/cdi-11-2017-0207

[B6] Bakker A. B. Demerouti E. (2014). Job demands-resources theory, in Work and wellbeing. Wellbeing: complete reference guide, eds Chen P. Y. Cooper C. L. (Hoboken, NJ :Wiley-Blackwell). 37–64. 10.1002/9781118539415.wbwell019

[B7] Balconi M. Fronda G. (2020). Morality and management: an oxymoron? fNIRS and neuromanagement perspective explain us why things are not like this. Cognit. Affect. Behav. Neurosci. 20, 1336–1348. 10.3758/s13415-020-00841-1 33123863 PMC7716886

[B8] Balconi M. Salati E. (2020). Dalle funzioni esecutive ai programmi di neuropotenziamento. Nuove prospettive per il neuroassessment, in Il neuromanagement tra cambiamento, tecnologia e benessere, eds Balconi M. Nava B. Salati E. (Milano: LED).

[B9] Bandura A. Schunk D. H. (1981). Cultivating competence, self-efficacy, and intrinsic interest through proximal self-motivation. J. Personal. Soc. Psychol. 41, 586–598. 10.1037/0022-3514.41.3.586

[B10] Barbier M. Hansez I. Chmiel N. Demerouti E. (2012): Performance expectations, personal resources, job resources: How do they predict work engagement? Eur. J. Work Organizational Psychol. 18, 1–13. 10.1080/1359432X.2012.704675

[B11] Bazzani A. Ravaioli S. Faraguna U. Turchetti G. (2020). Is EEG suitable for marketing research? A systematic review. Front. Neurosci. 14:594566. 10.3389/fnins.2020.594566 33408608 PMC7779633

[B12] Behrenz L. (2001). Who gets the job and why? An exploratory study of employers' recruitment behavior. J. Appl. Econ. 4, 255–278. 10.1080/15140326.2001.12040565

[B13] Berka C. Levendowski D. J. Lumicao M. N. Yau A. Davis G. Zivkovic V. T.. (2007). Eeg correlates of task engagement and mental workload in vigilance, learning, and memory tasks. Aviation Space Environ. Med. 78, B231–B244. 10.1177/1461445601003002004 17547324

[B14] Bilucaglia M. Laureanti R. Zito M. Circi R. Fici A. Rivetti F.. (2019). Looking through blue glasses: bioelectrical measures to assess the awakening after a calm situation, in 2019 41st Annual International Conference of the IEEE Engineering in Medicine and Biology Society (Piscataway, NJ: EMBC). 10.1109/EMBC.2019.8856486 31945953

[B15] Bolls P. D. Lang A. Potter R. F. (2001). The effect of message valence and listener arousal on attention, memory, and facial muscular responses to radio advertisements. Commun. Res. 28, 627–651. 10.1177/009365001028005003

[B16] Bonaccio S. Gauvin N. Reeve C. L. (2014). The experience of emotions during the job search and choice process among novice job seekers. J. Career Dev. 4, 237–257. 10.1177/0894845313486354

[B17] Borghini G. Di Flumeri G. Aric,ò P. Sciaraffa N. Bonelli S. Ragosta M.. (2020). A multimodal and signals fusion approach for assessing the impact of stressful events on Air Traffic Controllers. Sci. Rep. 10, 1–18. 10.1038/s41598-020-65610-z 32451424 PMC7248090

[B18] Boucsein W. Fowles D. C. Grimnes S. Ben-Shakhar G. Roth W. T. Dawson M. E.. (2012). Publication recommendations for electrodermal measurements. Psychophysiology 49, 1017–1034. 10.1111/j.1469-8986.2012.01384.x 22680988

[B19] Boudreau J. W. Boswell W. R. Judge T. A. Bretz R. D. (2001). Personality and cognitive ability as predictors of job search among employed managers. Person. Psychol. 54, 25–50. 10.1111/j.1744-6570.2001.tb00084.x

[B20] Cantele S. (2018). Human resources management in responsible small business: why, how and for what?'. Int. J. Hum. Res. Dev. Manage. 18, 237–257. 10.1504/IJHRDM.2018.10013652

[B21] Carless S. A. Imber A. (2007). The influence of perceived interviewer and job organizational characteristics on applicant attraction and job choice intention: the role of applicant anxiety. Int. J. Sel. Assess. 15, 359–371. 10.1111/j.1468-2389.2007.00395.x

[B22] Ciceri A. Russo V. Songa G. Gabrielli G. Clement J. (2019). A neuroscientific method for assessing effectiveness of digital vs. print ads. J. Advert. Res. 60, 71–86. 10.2501/jar-2019-015

[B23] Cocco G. C. (ed.). (2016). Neuromanagement. Per una nuova scienza del management. Milano: Franco Angel.

[B24] Consiglio C. Borgogni L. Tecco C. D. Schaufeli W. B. (2016). What makes employees engaged with their work? The role of self-efficacy and employee's perceptions of social context over time. Career Dev. Int. 21, 125–143. 10.1108/cdi-03-2015-0045

[B25] Cooper C. L. Marshall J. (1978). Understanding Esecutive Stress. London: Macmillian.

[B26] Davidson R. J. (2004). What does the prefrontal cortex “do” in affect: Perspectives on frontal eeg asymmetry research. Biological Psychology 67, 219–233. 10.1016/j.biopsycho.2004.03.008 15130532

[B27] Davies G. Mete M. Whelan S. (2018). When employer brand image aids employee satisfaction and engagement. J. Organizational Effect. 5, 64–80. 10.1108/joepp-03-2017-0028

[B28] De Vos A. De Stobbeleir K. Meganck A. (2009). The relationship between career-related antecedents and graduates' anticipatory psychological contracts. J. Business Psychol. 24, 289–298. 10.1007/s10869-009-9107-3

[B29] De Witte H. (1999). Job insecurity and psychological well-being: review of the literature and exploration of some unresolved issues. Eur. J. Work Organizational Psychol. 8, 155–177. 10.1080/135943299398302

[B30] Demerouti E. (2006). Job characteristics, flow, and performance: The moderating role of conscientiousness. J. Occupat. Health Psychol. 11, 266–280. 10.1037/1076-8998.11.3.266 16834474

[B31] Dries N. Pepermans R. De Kerpel E. (2008). Exploring four generations beliefs about career is satisfied the new successful?'. J. Manage. Psychol. 23, 907–928. 10.1108/02683940810904394

[B32] Emberland J. S. Rundmo T. (2010). Implications of job insecurity perceptions and job insecurity responses for psychological well-being, turnover intentions and reported risk behavior. Safety Sci. 48, 452–459. 10.1016/j.ssci.2009.12.002

[B33] Emmerling R. J. Cherniss C. (2003). Emotional intelligence and career choice process. J. Career Assess. 11, 153–167. 10.1177/1069072703011002003

[B34] Feiler A. R. Powell D. M. (2015). Behavioral expression of job interview anxiety. J. Business Psychol. 31, 155–171. 10.1007/s10869-015-9403-z

[B35] Frisch J. U. Häausser J. A. Mojzisch A. (2015). The trier social stress test as a paradigm to study how people respond to threat in social interactions. Front. Psychol. 6:14. 10.3389/fpsyg.2015.00014 25698987 PMC4313597

[B36] Frisch J. U. Hausser J. A. van Dick R. Mojzisch A. (2014). Making support work: the interplay between social support and social identity. J. Experi. Soc. Psychol. 55, 154–161. 10.1016/j.jesp.2014.06.009

[B37] Gabrielli G. Bilucaglia M. Zito M. Laureanti R. Caponetto A. Circi R.. (2020). Neurocoaching: exploring the relationship between coach and coachee by means of bioelectrical signal similarities, in 2020 42nd Annual International Conference of the IEEE Engineering in Medicine & Biology Society (EMBC) (Piscatawa, NJ). 10.1109/embc44109.2020.9176497 33018681

[B38] Gakhal B. Senior C. (2008). Examining the influence of fame in the presence of beauty: An electrodermal'neuromarketing' study. J. Consumer Behav. 7, 331–341. 10.1002/cb.255

[B39] Ghadiri A. Habermacher A. Peters T. (2012). Neuroleadership. Berlin: Springer.

[B40] Greene S. Thapliyal H. Caban-Holt A. (2016). A survey of affective computing for stress detection: Evaluating technologies in stress detection for better health. IEEE Consumer Electr. Magazine 5, 44–56. 10.1109/mce.2016.2590178

[B41] Hairston W. D. Whitaker K. W. Ries A. J. Vettel J. M. Bradford J. C. Kerick S. E.. (2014). Usability of four commercially-oriented EEG systems. J. Neural Eng. 11:046018. 10.1088/1741-2560/11/4/046018 24980915

[B42] Hallin K. Danielson E. (2008). Registered nurses' perceptions of their work and professional development. J. Adv. Nurs. 61, 62–70. 10.1111/j.1365-2648.2007.04466.x 18034817

[B43] Harmon-Jones E. Gable P. A. Peterson C. K. (2010). The role of asymmetric frontal cortical activity in emotion related phenomena: A review and update. Biol. Psychol. 8, 451–462. 10.1016/j.biopsycho.2009.08.010 19733618

[B44] Hatfield E. Cacioppo J. T. Rapson R. L. (1993). Emotional contagion. Curr. Direct. Psychol. Sci. 2, 96–100. 10.1111/1467-8721.ep10770953

[B45] Hobfoll S. E. Johnson R. J. Ennis N. Jackson A. P. (2003). Resource loss, resource gain, and emotional outcomes among inner city women. J. Personal. Soc. Psychol. 84, 632–643. 10.1037/0022-3514.84.3.632 12635922

[B46] Isen A. M. Reeve J. (2005). The influence of positive affect on intrinsic and extrinsic motivation: Facilitating enjoyment of play, responsible work behavior, and self-control. Motiv. Emot. 29, 297–325. 10.1007/s11031-006-9019-8

[B47] Jacobs S. C. Friedman R. Parker J. D. Tofler G. H. Jimenez A. H. Muller J. E.. (1994). Use of skin conductance changes during mental stress testing as an index of autonomic arousal in cardiovascular research. Am. Heart J. 128, 1170–1177. 10.1016/0002-8703(94)90748-x 7985598

[B48] Jamshed S. (2014). Qualitative research method-interviewing and observation. J. Basic Clin. Pharm. 5, 87–88. 10.4103/0976-0105.141942 25316987 PMC4194943

[B49] Jasper H. H. (1958). The ten-twenty electrode system of the international federation. Electroencephalogr. Clin. Neurophysiol. 10, 370–375 10590970

[B50] Karasek R. Brisson C. Kawakami N. Houtman I. Bongers P. Amick B. (1998). The job content questionnaire (jcq): an instrument for internationally comparative assessments of psychosocial job characteristics. J. Occupat. Health Psychol. 3, 322–355. 10.1037/1076-8998.3.4.322 9805280

[B51] Karasek R. A. Theorell T. (1990). Healthy Work: Stress, Productivity, and the Reconstruction of Working Life. New York, NY: Basic Books.

[B52] Keller K. L. (1993). Conceptualizing, measuring, and managing customer-based brand equity. J. Market. 57, 1–22. 10.2307/1252054

[B53] Keltner D. Gross J. J. (1999). Functional accounts of emotions. Cogn. Emotion 13, 467–480. 10.1080/026999399379140

[B54] Kerse G. Kocak D. Ozdemir S. (2018). Does the perception of job insecurity bring emotional exhaustion? the relationship between job insecurity, affective commitment and emotional exhaustion. Busin. Econo. Res. J. 9, 651–663. 10.20409/berj.2018.129

[B55] Kidd J. M. (1998). Emotion: An absent presence in career theory. J. Vocat. Behav. 52, 275–288. 10.1006/jvbe.1997.1629

[B56] Laszlo K. D. Pikhart H. Kopp M. S. Bobak M. Pajak A. Malyutina S.. (2010). Job insecurity and health: a study of 16 european countries. Soc. Sci. Med. 70, 867–874. 10.1016/j.socscimed.2009.11.022 20060634 PMC2845821

[B57] Laureanti R. Bilucaglia M. Zito M. Circi R. Fici A. Rivetti F.. (2020). Emotion assessment using machine learning and low-cost wearable devices, in 2020 42nd Annual International Conference of the IEEE Engineering in Medicine & Biology Society (EMBC) (Piscataway, NJ). 10.1109/EMBC44109.2020.9175221 33018054

[B58] Liapis A. Katsanos C. Sotiropoulos D. Xenos M. Karousos N. (2015). Recognizing emotions in human computer interaction: Studying stress using skin conductance. Human-Comp. Interact. 2015, 255–262. 10.1007/978-3-319-22701-618

[B59] Love J. Selker R. Marsman M. Jamil T. Dropmann D. Verhagen J.. (2019). Jasp: Graphical statistical software for common statistical designs. J. Statist. Softw. 88, 1–17. 10.18637/jss.v088.i02

[B60] Ma Q. Wang X. (2006). Cognitive neuroscience, neuroeconomics, and neuromanagement. Manage. World 10, 139–149.

[B61] Ma Q. Wang X. Dai S. Shu L. (2007). Event-related potential n270 correlates of brand extension. Neuroreport 18, 1031–1034. 10.1097/WNR.0b013e3281667d59 17558290

[B62] Manuti A. Giancaspro M. L. Molino M. Ingusci E. Russo V. Signore F.. (2020). “Everything will be fine”: A study on the relationship between employees' perception of sustainable HRM practices and positive organizational behavior during COVID19. Sustainability 12:10216. 10.3390/su122310216

[B63] Mason J. (1994). Linking Qualitative and Quantitative Data Analysis. Analysing Qualitative Data. London: Routledge.

[B64] McCarthy J. Goffin R. (2004). Measuring job interview anxiety: beyond weak knees and sweaty palms. Person. Psychol. 57, 607–637. 10.1111/j.1744-6570.2004.00002.x

[B65] Melchers K. G. Bösser D. Hartstein T. Kleinmann M. (2012). Assessment of situational demands in a selection interview: reflective style or sensitivity? Int. J. Select. Assess. 20, 475–585. 10.1111/ijsa.12010

[B66] Missaglia A. L. Oppo A. Mauri M. Ghiringhelli B. Ciceri A. Russo V. (2017). The impact of emotions on recall: an empirical study on social ads. J. Consu. Behav. 16, 1–10. 10.1002/cb.1642

[B67] Parincu A. M. T. (2019). Neuromanagement & the impact of neuroscience on the organizational performance. Risk Contemp. Econ. 487–493. 10.35219/rce2067053256

[B68] Parincu A. M. T. Capatina A. Varon D. J. Bennet P. F. Recuerda A. M. (2020). Neuromanagement: the scientific approach to contemporary management. Proc. Int. Conf. Business Excel. 14, 1046–1056. 10.2478/picbe-2020-0099

[B69] Passyn K. Sujan M. (2006). Self-accountability emotions and fear appeals: Motivating behavior. J. Consumer Res. 32, 583–589. 10.1086/500488

[B70] Plassmann H. Ramsøy T. Milosavljevic M. (2012). Branding the brain: a critical review and outlook. J. Consumer Psychol. 22, 18–36. 10.1016/j.jcps.2011.11.010

[B71] Plassmann H. Venkatraman V. Huettel S. Yoon C. (2015). Consumer neuroscience: applications, challenges, and possible solutions. J. Market. Res. 52, 427–435. 10.1509/jmr.14.0048

[B72] Poels K. DeWitte S. (2006). How to capture the heart? reviewing 20 years of emotion measurement in advertising. J. Advert. Res. 46, 18–37. 10.2501/S0021849906060041

[B73] Posada-Quintero H. F. Chon K. H. (2020). Innovations in electrodermal activity data collection and signal processing: A systematic review. Sensors 20:479. 10.3390/s20020479 PMC7014446 31952141

[B74] Powell D. M. Stanley D. J. Brown K. N. (2018). Meta-analysis of the relation between interview anxiety and interview performance. Canad. J. Behav. Sci. 50, 195–207. 10.1037/cbs0000108posada-

[B75] Raccanello D. (2015). Students' expectations about interviewees' and interviewers' achievement emotions in job selection interviews. J. Employ. Counsel. 52, 50–64. 10.1002/joec.12004

[B76] Ravaja N. (2004). Contributions of psychophysiology to media research: review and recommendations. Med. Psychol. 6, 193–235. 10.1207/s1532785xmep06024

[B77] Reznik S. J. Allen J. J. (2018). Frontal asymmetry as a mediator and moderator of emotion: An updated review. Psychophysiology 55:12965. 10.1111/psyp.12965 28776710

[B78] Rothschild M. Hyun Y. J. (1990). Predicting memory for components of tv commercials from eeg. J. Consumer Res. 16, 472–478. 10.1086/209232

[B79] Russo V. Milani Marin L. E. Fici A. Bilucaglia M. Circi R. Rivetti F.. (2021). Strategic communication and neuromarketing in the fisheries sector: generating ideas from the territory. Front. Commun. 6:49. 10.3390/socsci9070123

[B80] Russo V. Valesi R. Gallo A. Laureanti R. Zito M. (2020). “the theater of the mind”: The effect of radio exposure on tv advertising. Soc. Sci. 9:123. 10.3389/fcomm.2021.659484

[B81] Salanova M. Schaufeli W. B. Xanthopoulou D. Bakker A. B. (2010). The gain spiral of resources and work engagement: Sustaining a positive worklife, in Work Engagement: A Handbook of Essential Theory and Research, eds Bakker A. B. Leiter M. P. (New York, NY: Psychology Press).

[B82] Sandhya S. Sulphey M. (2019). An assessment of contribution of employee engagement, psychological contract and psychological empowerment towards turnover intentions of IT employees. Int. J. Environ. Workplace Employ. 5:22. 10.1504/ijewe.2019.097186

[B83] Satpathy J. (2012). Issues in neuro - management decision making. Opinion 2, 23–36.

[B84] Schaufeli W. B. Bakker A. B. (2010). The conceptualization and measurement of work engagement: A review, in Work Engagement: A Handbook of Essential Theory and Research, eds Bakker A. B. Leiter M. P. (Hove; East Sussex: Psychology Press).

[B85] Scheuer J. (2001). Recontextualization and communicative styles in job interviews. Discourse Stud. 3, 223–248.

[B86] Schneider B. Bowen D. E. (1993). The service organization: human resources management is crucial. Organizational Dyn. 21, 39–52. 10.1016/0090-2616(93)90032-V

[B87] Schneider B. Smith D. B. Paul M. C. (2001). P-e fit and the attraction-selection attrition model of organizational functioning: Introduction and overview, in Work Motivation in the Context of a Globalizing Economy, eds Erez M. Kleinbeck U. Thierr H. (London: Erlbaum).

[B88] Sequeira H. Hot P. Silvert L. Delplanque S. (2009). Electrical autonomic correlates of emotion. Int. J. Psychophysiol. 71, 50–56. 10.1016/j.ijpsycho.2008.07.009 18723054

[B89] Setti I. Zito M. Colombo L. Cortese C. G. Ghislieri C. Argentero P. (2018). Well-being and affective commitment among ambulance volunteers: a mediational model of job burnout. J. Soc. Serv. Res. 44, 236–248. 10.1080/01488376.2018.1442898

[B90] Sieverind M. Weidner G. von Volkmann B. (2005). Cardiovascular reactivity in a simulated job interview: The role of gender role self-concept. Int. J. Behav. Med. 12, 1–10. 10.1207/s15327558ijbm12011 15743730

[B91] Subramanian S. Barbieri R. Brown E. N. (2019). A systematic method for preprocessing and analyzing electrodermal activity, in 2019 41st Annual International Conference of the IEEE Engineering in Medicine and Biology Society (EMBC). Piscatawa, NJ: IEEE. 10.1109/EMBC.2019.8857757 31947426

[B92] Sverke M. Hellgren J. (2002). The nature of job insecurity: Understanding employment uncertainty on the brink of a new millennium. Appl. Psychol. 51, 23–42. 10.1111/1464-0597.0077z

[B93] Tanner R. J. Ferraro R. Chartrand T. Y. Bettman J. R. Van Baaren R. (2008). Of chameleons and consumption: The impact of mimicry on choice and preferences. J. Consumer Res. 34, 754–766. 10.1086/522322

[B94] Tay C. Ang S. Van Dyne L. (2006). Personality, biographical characteristics, and job interview success: A longitudinal study of the mediating effects of interviewing self-efficacy and the moderating effects of internal locus of causality. J. Appl. Psychol. 91, 446–454. 10.1037/0021-9010.91.2.446 16551195

[B95] van de Bos R. Taris R. Scheppink B. de Haan L. Verster J. C. (2014). Salivary cortisol and alphaamylase levels during an assessment procedure correlate differently with risk-taking measures in male and female police recruits. Front. Behav. Neurosci. 7:219. 10.3389/fnbeh.2013.00219 24474909 PMC3893681

[B96] Van Voorhis C. R. W. Morgan B. L. (2007). Understanding power and rules of thumb for determining sample sizes. Tutor. Quant. Methods Psychol. 3, 43–50. 10.20982/tqmp.03.2.p043

[B97] Venturella I. Gatti L. Vanutelli M. E. Balconi M. (2017). When brains dialogue by synchronized or unsynchronized languages. Hyperscanning applications to neuromanagement. Neuropsychol. Trends 21, 35–51. 10.7358/neur-2017-021-vent

[B98] Verma J. P. (2015). Repeated Measures Design for Empirical Researchers. Hoboken, NJ: John Wiley & Sons.

[B99] Witte H. D. Elst T. V. Cuyper N. D. (2015). Job Insecurity, Health and Well-Being. Aligning Perspectives on Health, Safety and Well-Being. New York, NY: Springer. 10.1007/978-94-017-9798-67

[B100] Xanthopoulou D. Bakker A. B. Fischbach A. (2013). Work engagement among employees facing emotional demands: The role of personal resources. J. Person. Psychol. 12, 74–84. 10.1027/1866-5888/a000085

[B101] Yarkoni T. Poldrack R. A. Nichols T. E. Van Essen D. C. Wager T. D. (2011). Large-scale automated synthesis of human functional neuroimaging data. Nat. Methods 8, 665–670. 10.1038/nmeth.1635 21706013 PMC3146590

[B102] Zito M. Cortese C. G. Colombo L. (2016). Nurses' exhaustion: the role of flow at work between job demands and job resources. J. Nurs. Manage. 24, E12–22. 10.1111/jonm.12284 25612156

[B103] Zito M. Cortese C. G. Colombo L. (2019). The role of resources and flow at work in well-being. Sage Open 9, 1–12. 10.1177/215824401984973234290901

